# Development of molecular markers for invasive alien plants in Korea: a case study of a toxic weed, *Cenchrus longispinus* L., based on next generation sequencing data

**DOI:** 10.7717/peerj.7965

**Published:** 2019-11-11

**Authors:** JongYoung Hyun, Hoang Dang Khoa Do, Joonhyung Jung, Joo-Hwan Kim

**Affiliations:** Department of Life Science, Gachon University, Seongnam, Gyeonggi, Korea

**Keywords:** *Cenchrus longispinus*, *Cenchrus echinatus*, Invasive alien plants, Chloroplast genome, Molecular markers

## Abstract

**Background:**

Genomic data play an important role in plant research because of its implications in studying genomic evolution, phylogeny, and developing molecular markers. Although the information of invasive alien plants was collected, the genomic data of those species have not been intensively studied.

**Methods:**

We employ the next generation sequencing and PCR methods to explore the genomic data as well as to develop and test the molecular markers.

**Results:**

In this study, we characterize the chloroplast genomes (cpDNA) of *Cenchrus longispinus* and *C. echinatus*, of which the lengths are 137,144 and 137,131 bp, respectively. These two newly sequenced genomes include 78 protein-coding genes, 30 tRNA, and four rRNA. There are 56 simple single repeats and 17 forward repeats in the chloroplast genome of *C. longispinus*. Most of the repeats locate in non-coding regions. However, repeats can be found in *infA, ndhD, ndhH, ndhK, psbC, rpl22, rpoC2, rps14, trnA-UGC, trnC-GCA, trnF-GAA, trnQ-UUG, trnS-UGA, trnS-GCU*, and *ycf15*. The phylogenomic analysis revealed the monophyly of *Cenchrus* but not *Panicum* species in tribe Paniceae. The single nucleotide polymorphism sites in *atpB, matK*, and *ndhD* were successfully used for developing molecular markers to distinguish *C. longispinus* and related taxa. The simple PCR protocol for using the newly developed molecular markers was also provided.

## Introduction

*Cenchrus* is a member of 780 genera of Poaceae and has widespread distributions in Asia, Africa, Australia, and America (Govaerts, 2011; Christenhusz & Byng, 2016). *Cenchrus longispinus*, also called long-spined sandbur or spiny burr grass, is native to North America. However, this species invaded into other continents and became a noxious weed (Strat, Stoyanov & Holobiuc, 2017; Fagaras, 2018). In Korea, *C. longispinus* has been recognized as one of 320 invasive alien plants, which locates in Incheon province (Jung et al., 2017). Among *Cenchrus* species, *C. longispinus* and *C. echinatus* share the features of ovoid to globose burs with longer inner bristkes, sparse or numerous outer bristles, and the mostly fused or half fusion of the inner bristles (Verloove & Sánchez Gullón, 2012). However, *C. echinatus* is distinguished from *C. longispinus* by the characters of the burs which have numerous flexible and distinctly restrorsely barbellate outer bristles, and a single whorl inner bristles that form flattened spines. Previously, various studies on the management of *C. longispinus* have been conducted (Anderson, 1997; Soltani et al., 2010). Although the invasion and control of *C. longispinus* have been conducted, the study on its genomic data has not been approached. However, the genomic data of other *Cenchrus* (i.e., the chloroplast genome of *C. purpureus* and *C. ciliaris*) were reported (Bhatt & Thaker, 2018; Wu & Zhou, 2018). Chloroplast genome (cpDNA) plays an important role in plants because it contains essential genes for performing photosynthesis (Sugiura, 1992). Additionally, the highly conserved of cpDNA among plants resulted in the useful applications of cpDNA data in reconstructing phylogeny, exploring biogeography, surveying population genetics, and developing molecular markers (Cui et al., 2017; Fischer et al., 2017; Leaché & Oaks, 2017; Pantoja et al., 2017). In Poaceae, different studies on cpDNA have been reported (Matsuoka et al., 2002; Hand et al., 2013; Zhang et al., 2016; Huang et al., 2017). Specifically, there are different inversion event in cpDNA of the grass family (Doyle et al., 1992). Additionally, loss of gene (i.e., *accD*) was also found in the cpDNA of Poaceae (Huang et al., 2017). Also, the usage of cpDNA for phylogeny and development of molecular marker have been conducted among Poaceae species (Zhang, 2000; Soreng et al., 2017; Saarela et al., 2018).

The genomic data provide a profound understanding of the evolution and the potential solution for management of the invasive alien plants. Although the list of invasive alien plants in Korea was published, further studies on the management and genomic data have not been conducted. Therefore, in this study, we based on the next generation sequencing method to (1) complete and characterize the chloroplast genomes of *C. longispinus* and *C. echinatus*, (2) reconstruct the phylogeny of *Cenchrus* and related taxa in tribe Paniceae, and (3) develop molecular markers inferred from the single nucleotide polymorphisms (SNP) sites in *atpB, matK*, and *ndhD* to distinguish *C. longspinus* in Korea from related taxa.

## Materials and Methods

### Taxon sampling, DNA extraction, chloroplast genome assembly, and comparison

The fresh leaves of *C. longispinus* and *C. echinatus* were collected in Daecheong Island (Incheon, South Korea) and Texas (USA), respectively. These samples were dried using silica gel powder before their total DNA was extracted based on the modified CTAB method (Doyle & Doyle, 1987). The high-quality DNA (>200 ng/1 µl) was used for constructing NGS data through Miseq platform (Illumina, Seoul, Korea). The complete chloroplast genome of *C. purpureus* (Accession number MF594682) was used as a reference genome for the assembly of cpDNA of *C. longispinus* and *C. echinatus*. After the NGS data were imported into Geneious program (Kearse et al., 2012), only reads that have over 95% similarity to reference cpDNA were isolated. Then, the isolated reads were assembled to complete the cpDNA sequences using *denovo* assemble function in Geneious. Consequently, 95,386 out of 5,053,948 reads (1.9%) were assembled to complete the cpDNA of *C. longispinus* (Accession number MN078361) with the coverage of 208×. In the case of *C. echinatus*, only 20,267 out of 4,211,108 reads built up the complete cpDNA (Accession number MN078360) with the coverage of 44.3×. The complete cpDNA sequences of *C. longispinus* and *C. echinatus* were annotated in Geneious based on the previously published cpDNA of *C. purpureus*. The annotations that have over 95% similarity were kept and manually checked and adjusted the start and stop codons. The tRNA sequences were checked using tRNA Scan-SE (Chan & Lowe, 2019). The map of cpDNA was illustrated using OGDraw (Greiner, Lehwark & Bock, 2019). The complete cpDNA sequences of *Cenchrus* and related taxa were aligned using MAUVE embedded in Geneious (Darling et al., 2004). The REPuter program was used to locate the repeats in the chloroplast genomes with the minimum repeat length of 18 bp (Kurtz et al., 2001). To analyze the simple sequence repeat (SSR), the Phobos program was used with the setting of minimum repeated time of ten, five, four, three, and three for mono-, di-, tri-, tetra-, and pentanucleotide repeats, respectively (Christoph, 2006–2010).

### Phylogenomic analysis

The complete cpDNA sequences of *Cenchrus* and related taxa were downloaded from NCBI (Table S1). The 75 protein-coding regions of cpDNA were extracted and aligned using MUSCLE embedded in Geneious (Edgar, 2004). Additionally, three partition data sets (including large single copy (LSC), small single copy (SSC), and inverted repeat (IR) regions) were used to test the phylogenetic utility of different regions in cpDNA of *Cenchrus* and related species. Three regions were also aligned using MUSCLE embedded in Geneious. Then, the aligned sequences were imported to jModeltest to find the best model for elucidating the phylogeny of *Cenchrus* (Posada, 2008). Consequently, the TVM+I+G model was selected as the best model for further analysis. The Maximum likelihood (ML) and Bayesian Inference (BI) analysis were conducted using IQtree (Trifinopoulos et al., 2016) and MrBayes (Ronquist et al., 2012). In the ML analysis, the bootstrapping test was repeated 1,000 times for calculating bootstrap values. Meanwhile, the BI analysis was run with one million generations. The phylogenetic tree was illustrated using FigTree (http://tree.bio.ed.ac.uk/software/figtree/).

### Development of molecular markers for *Cenchrus longispinus*

**Figure 1:**
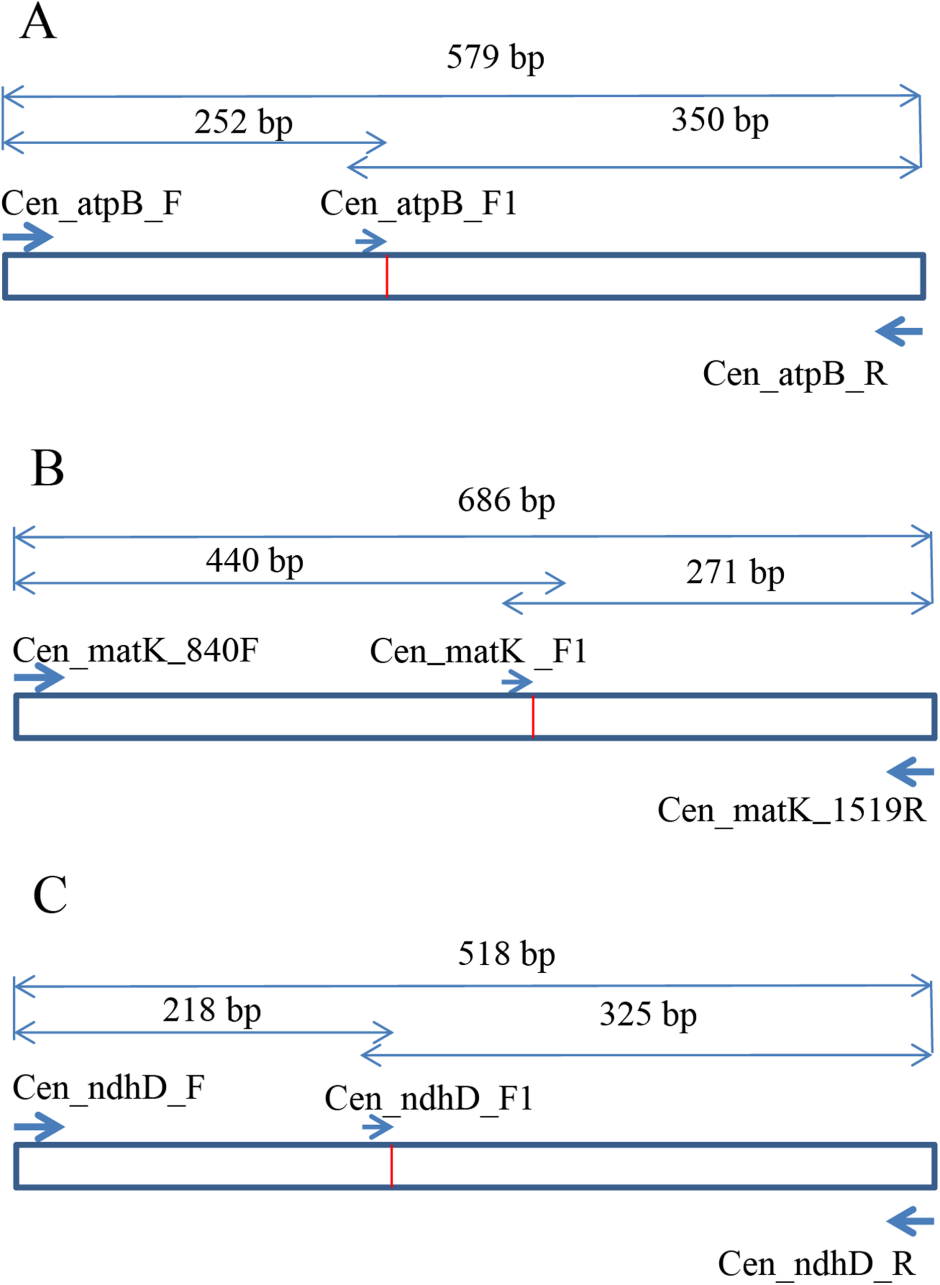
The design of primer pairs for SNP sites in *Cenchrus longispinus*. (A) The primer pairs in the *atpB* gene; (B) the primer pairs in the *matK* gene; (C) the primer pairs in the *ndhD* gene.

To develop the molecular marker for *C. longispinus*, the cpDNA sequences of *C. longispinus* and *C. echinatus* were aligned to locate the SNP sites. The primer pairs that cover the regions containing SNP sites were designed based on the conserved regions in two *Cenchrus*. Then, these primer pairs were used for *Pennisetum alopecuroides*, which has a close relationship to *Cenchrus* and has a wide distribution in Korea. The primer pairs that resulted in PCR products in *Pennisetum* sample were selected for sequencing. The new sequences from *Pennisetum* were aligned with those of *Cenchrus* to locate the specific SNP sites for *C. longispinus*. Consequently, the *atpB*, *matK*, and *ndhD* were selected to develop the molecular markers for *C. longispinus*. The primer pairs for three genes were designed using Primer3 (Untergasser et al., 2012; Fig. 1; Table S2). The specific lengths of PCR products were designed for *C. longispinus* (Fig. 1). The PCR mixture and protocol were described in Table S3. For testing the efficiency of the primer pairs, further samples of three species were collected from different locations with the permission from Korea National Arboretum (KNA), Gachon University Herbarium (GCU); The University of Texas at Austin Herbarium (TEX), and The Queensland Herbarium (BRI) (Table S4). The PCR products were checked using agarose gel 1% and electrophoresis method.

## Results

### Plastome features of *Cenchrus*

**Figure 2:**
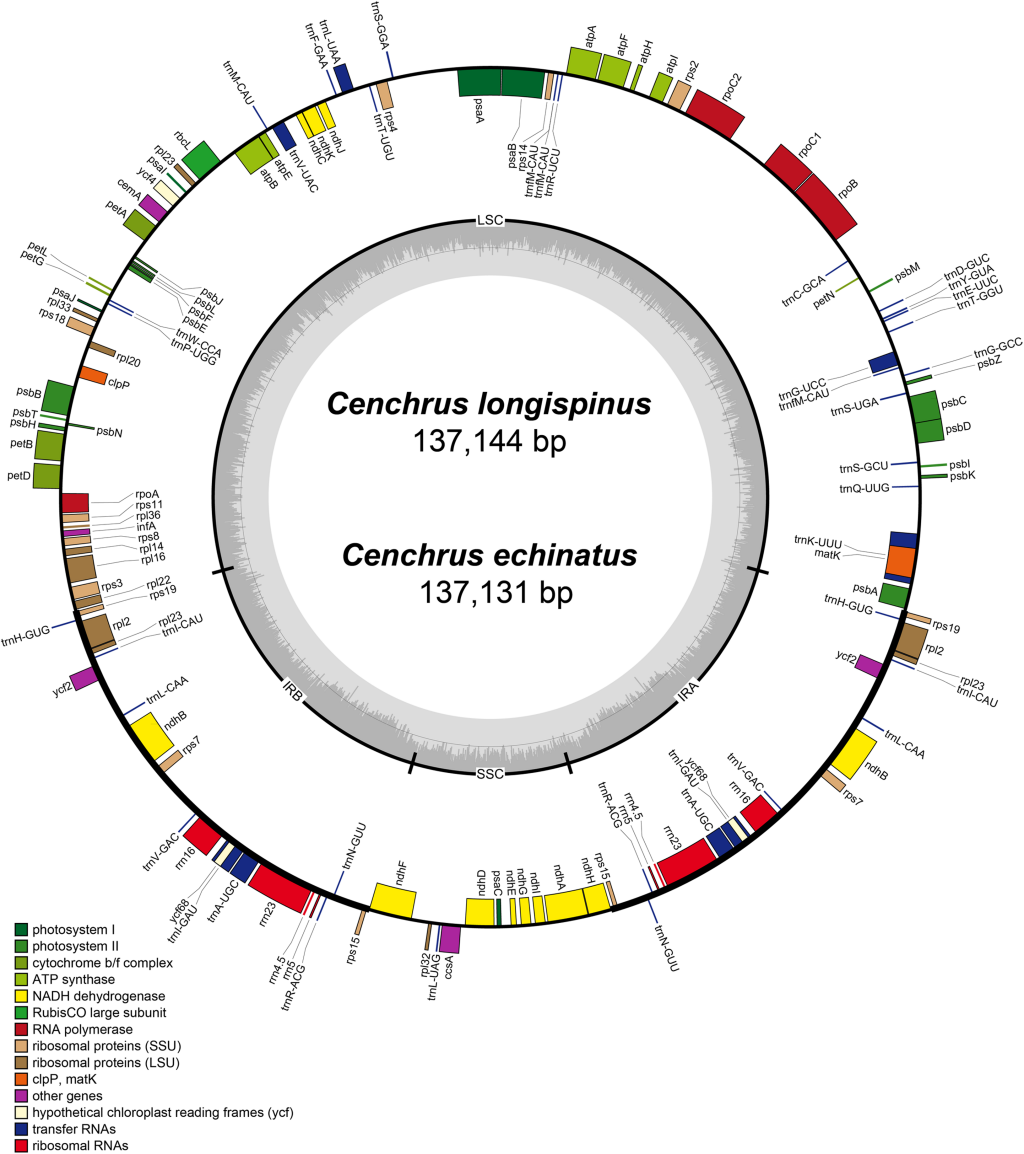
The map of chloroplast genomes of *Cenchrus*. The genes inside of the circle are transcribed clockwise, whereas the genes outside of the circle are transcribed counterclockwise. LSC, large single copy; SSC, small single copy; IRA-IRB, inverted repeat regions.

The complete cpDNA of *C. longispinus* has a quadripartite structure which consists of LSC region of 80,223 bp, a SSC region of 12,449 bp and two inverted repeat regions of 22,236 bp (Table 1; Fig. 2). A similar structure was also found in cpDNA of *C. echinatus*; however, the lengths of LSC, SSC, and IR regions are 80,220, 12,439, and 22,236 bp, respectively. Although the lengths of LSC and SSC regions are different, the sizes of IR regions are identical in three examined *Cenchrus*. In comparison with related taxa, there is no identical length of three regions (Table 1). Albeit the lengths of three regions are various, the number of protein-coding genes (79), tRNA (30). and rRNA (4) are identical among *Cenchrus* and surveyed species (Table 1; Table S5), except *Panicum capillare* which do not have *ycf2* and *ycf15* regions. Among the three examined *Cenchrus*, *C. longispinus* is more similar to *C. echinatus* (99.9% pairwise identity) than *C. purpureus* (97.3% pairwise identity). Compared to other examined species, the pairwise similarity of *C. longispinus* cpDNA is less than 95% (Table 1). The IR-LSC junction locates in the intergenic space (IGS) between *rps19* and *rpl22*, whereas the SSR-IR border is in the *ndhF* coding region of *Cenchrus* and related taxa (Table 1). There are 56 SSRs and 17 forward repeats in the chloroplast genome of *C. longispinus* (Tables S6 and S7). Most of SSRs are composed of A and T nucleotides. Additionally, locations of these SSRs are in non-coding regions, except ten SSRs that were found in *infA, ndhD, ndhH, ndhK, rps14, rpl22, rpoC2, psbC*, and *ycf5* (Table S6). Similarly, 17 forward repeats locate in both non-coding (13 sites) and coding regions (five sites, Table S7). Although *C. longispinus* is highly similar to *C. echinatus*, the number and length of repeats are different between two species (Tables S6 and S7).

**Table 1:** Features of Chloroplast genomes and pairwise identity among *Cenchrus* species and related taxa.

Species	*Cenchrus longispinus*	*C. echinatus*	*C. purpureus*	*Eriochloa meyeriana*	*Panicum capillare*	*Dichanthelium acuminatum*	*Thyridolepsis xerophila*	*Lecomtella madagascariensis*
Accession number	MN078361	MN078360	MF594682	KU291498	KU291475	KU291496	KU291485	HF543599
Total length (bp)	137,144	137,131	138,199	139,890	134,520	140,122	140,644	139,073
GC content (%)	38.7	38.7	38.6	38.4	38.5	38.7	38.7	38.6
Lagre single copy (LSC-bp)	80,223	80,220	81,161	81,856	81,858	82,065	82,641	81,349
Small single copy (SSC-bp)	12,449	12,439	12,386	12,568	12,576	12,617	12,599	12,452
Inverted repeat (IR-bp)	22,236	22,236	22,236	22,733	20,043	22,720	22,702	22,636
Protein coding genes	78	78	78	78	76	78	78	78
tRNA	30	30	30	30	30	30	30	30
rRNA	4	4	4	4	4	4	4	4
LSC-IR junction	IGS (*rps19-rpl22*)	IGS (*rps19-rpl22*)	IGS (*rps19-rpl22*)	IGS (*rps19-rpl22*)	IGS (*rps19-rpl22*)	IGS (*rps19-rpl22*)	IGS (*rps19-rpl22*)	IGS (*rps19-rpl22*)
SSC-IR junction	*ndhF* (29 bp)	*ndhF* (29 bp)	*ndhF* (29 bp)	*ndhF* (29 bp)	*ndhF* (29 bp)	*ndhF* (29 bp)	*ndhF* (29 bp)	*ndhF* (17 bp)
Pairwise Identity (%)	100	99.9	97.3	94.6	92.3	94.4	94.9	94.4

### Phylogenomics analysis of *Cenchrus* and related taxa

**Figure 3:**
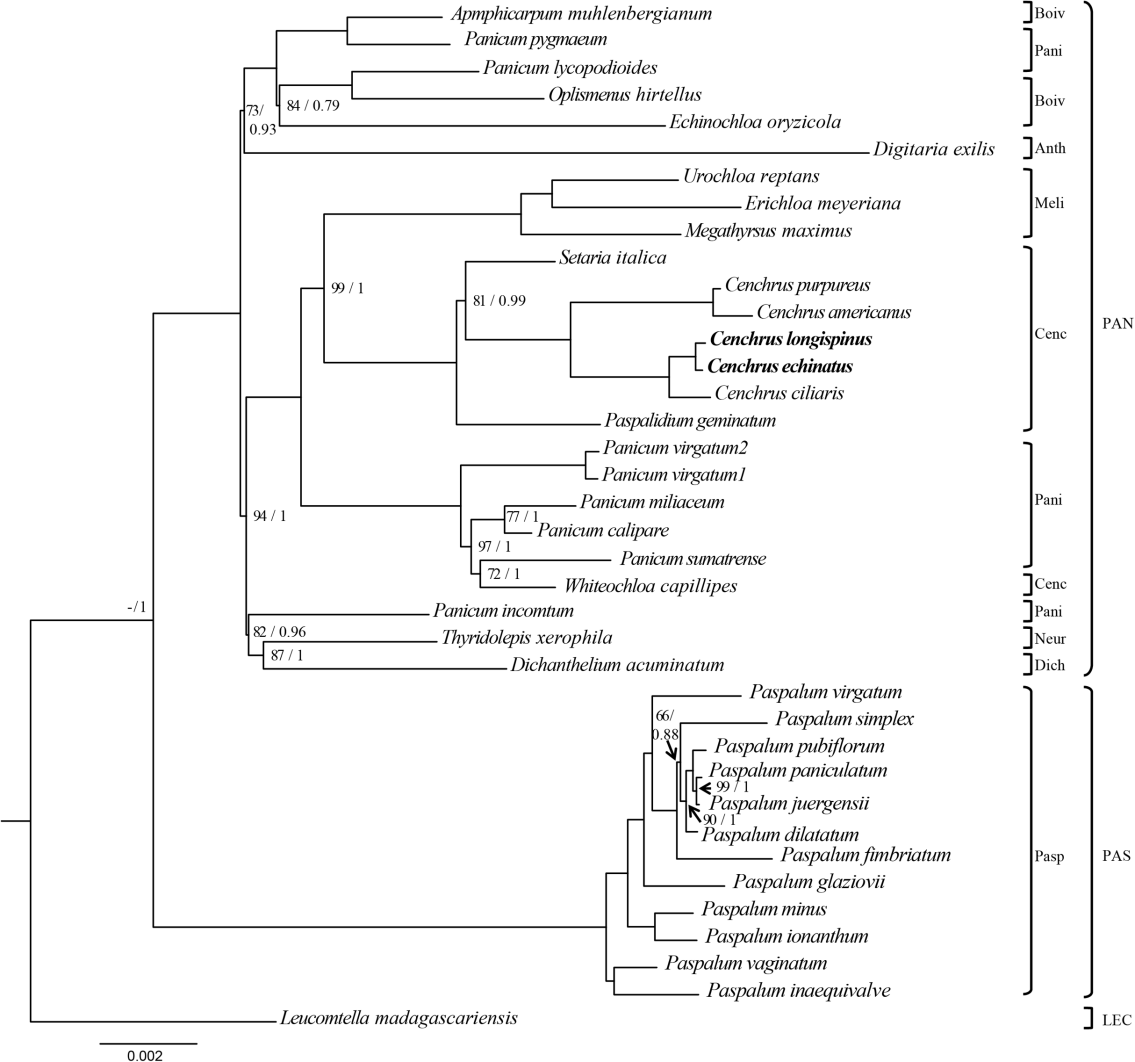
The Maximum likelihood tree of *Cenchrus* and related taxa inferred from 75 chloroplast genes. The numbers mean supporting values (Bootstrap (BP)/ Posterior probability (PP)). Only supporting values under (BP = 100/PP = 1) were shown. The dash means no value. LEC, Lecomtelleae; PAN, Paniceae; PAS, Paspaleae; Pasp, Paspalinae; Anth, Anthephorinae; Boiv, Boivinellinae; Neur, Neurachninae; Dich, Dichantheliinae; Pani, Panicinae; Meli, Melinidinae; Cenc, Cenchrinae.

The ML and BI analysis generated the same topology of phylogenetic trees, of which the monophyly of *Cenchrus* was revealed with high supporting value (Fig. 3). Although the tribe Paniceae is monophyletic, some subtribes are polyphyletic. Specifically, *Whiteochloa capillipes* located within subtribe Paniceae although it is a member of subtribe *Cenchrinae*. Another polyphyletic group is subtribe Panicinae of which *Panicum pygmaeum* and *Panicum lycopodioides* from a clade with the species of subtribe Boivinellinae. Additionally, *Panicum incomtum* is sister to *Thyridopepsis xerophila* (subtribe Neurachninae) and *Dichanthelium acuminatum* (subtribe Dichantheliinae). The three partition data (LSC, SSC, and IR regions) resulted in different topologies of phylogenetic trees (Figs. S2–S4). However, the relationships of *Cenchrus* and related taxa inferred from LSC and SSC region data sets are quite similar to those of 75 protein-coding genes, except for *Paspalidium geminatum* and *Digitaria exilis*. In contrast, the IR region-based phylogenetic tree exhibits different relationships in tribe Paniceae compared to other data sets (Fig. S3). However, the monophyly of *Cenchrus* was found in all data sets with strong supporting values.

### Specific SNP-based molecular markers for *Cenchrus longispinus*

**Figure 4:**
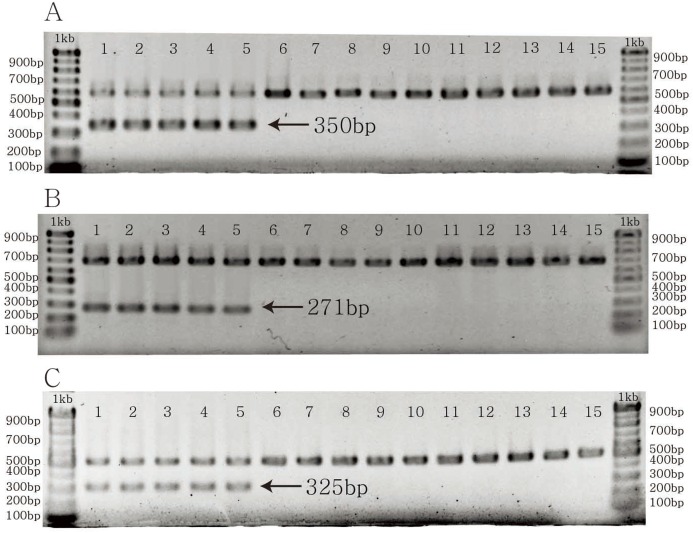
The PCR results of specific primer pairs for *Cenchrus longispinus*. (A) The specific primer pairs for *atpB* gene; (B) the specific primer pairs for *matK* gene; (C) the specific primer pairs for *ndhD* gene. The number from one to five: *Cenchrus longispinus*; from six to 10: *Cenchrus echinatus*; from 11 to 15: *Pennisetum alopecuroides*.

The specific primer pairs for *C. longispinus* inferred from *atpB, matK*, and *ndhD* resulted in bands of 350, 271, and 325 bp, respectively (Fig. 4). There are no bands for *C. echinatus* and *Pennisetum alopecuroides*, except the control bands of 579, 686, 518 bp from *atpB*, *matK*, and *ndhD*, respectively. The primer pairs are also effective in other examined species from Korea, the USA, and other countries (Table S4; Fig. S1).

## Discussion

### The chloroplast genomes of *Cenchrus* and phylogenomic implication

The chloroplast genomes of *Cenchrus* are highly conserved regards structure, gene content and order in comparison with other species in tribe Paniceae (Table 1). Previously, the inversion event of 28 kb-region was reported in the grass family (Doyle et al., 1992). Additionally, Huang et al. (2017) suggested a model for rearrangement of LSC in the Poaceae. The process includes two inversion events of which the first inversion of 28 kb-region was followed by a second inversion between *trnS-GCU* and *trnT-GGU*. Besides inversions, the loss of intron was also recorded in Poaceae (Huang et al., 2017). In *Cenchrus* and other members of tribe Paniceae, the cpDNA also have the inversions and loss of intron in *rpoC1* (Table 1).

In the chloroplast genome, the IR regions can be contracted or expanded, which resulted in various boundaries among LSC, SSC, and IR regions of different green plants (Wang et al., 2008; Downie & Jansen, 2015; Wang et al., 2015; Raman & Park, 2016; Cauz-Santos et al., 2017). Notably, the loss of IR region was found to be reversed in legumes (Choi, Jansen & Ruhlman, 2019). In the previously published cpDNA of Poaceae, the loss of IR regions was not found and there are different LSC-IR-SSC junctions. However, the IR-LSC junction is quite stable, whereas there is more variable in SSC-IR boundary (Huang et al., 2017). Both IR-LSC junction and SSC-IR border are stable at the tribal and genus level in Poaceae. For example, in tribe Oryzeae, the IR-LSC junction is in the IGS between *rpl22* and *rps19* whereas the SSC-IR border is in the coding region of *ndhH* (Zhang et al., 2016). A similar trend was also found in *Festuca* and *Lolium* (tribe Poeae; Qiu et al., 2019). In this study, the junctions among *Cenchrus* and related taxa in tribe Paniceae are similar (the IR-LSC and IR-SSC junctions in the IGS of *rps19- rpl22*, and the *ndhF*, respectively), which provides more evidence to support the structural stability of junctions at tribal and genus level in Poaceae (Table 1). However, the available complete cpDNA have not covered all tribes and genera in Poaceae. Also, the study in evolution of chloroplast genomes, which includes the representatives of all subfamilies, tribes, and genera in Poaceae, has not been conducted. Therefore, further studies that cover more samples in Poaceae should be conducted to trace the structural evolution of chloroplast genomes in Poaceae, an ecologically and economically important family among green plants.

The phylogenomic analysis inferred from 75 protein-coding regions highly support the monophyly of *Cenchrus* (Fig. 3). Previously, different studies revealed the close relationship between *Cenchrus* and *Pennisetum* and the merge of *Pennisetum* to *Cenchrus* was also suggested (Chemisquy et al., 2010; Veldkamp, 2014). In this study, the relationship between *Pennisetum* and *Cenchrus* was not checked due to the lack of complete cpDNA sequences of *Pennisetum*. Therefore, further studies on the comparative genomics of these two genera should be conducted to give a better understanding of their evolutionary histories in Poaceae.

In contrast to the monophyly of *Cenchrus*, *Panicum* species is polyphyletic (Fig. 3). In previous studies, *Panicum* is sister to *Whiteochloa capillipes* (Burke et al., 2016; Saarela et al., 2018). As a result, Saarela et al. (2018) suggested transferring *Whiteochloa* from subtribe Cenchrinae to subtribe Panicinae. In this study, *Panicum* species forms a clade with not only *Whiteochloa* but also other species in subtribes Boivinellinae, Neurachninae, and Dichantheliinae (Fig. 3). Zuloaga, Salariato & Scataglini (2018) provided a deeper understanding of the phylogeny of *Panicum* and suggested a new circumscription of *Panicum*, including seven sections, based on *ndhF* data. However, the sample of *Panicum incomtum* was not included in their data. In the present study, the ML and BI analysis revealed a close relationship between *Panicum incomtum, Thyridopepsis xerophila* (subtribe Neurachninae), and *Dichanthelium acuminatum* (subtribe Dichantheliinae, Fig. 3), which was not shown in the study of Zuloaga, Salariato & Scataglini (2018). Therefore, more samples and molecular data of *Panicum* should be covered to clarify its relationship to other members of tribe Panicinae in further studies.

Previously, partition data inferred from LSC, SSC, and IR regions were used to test the phylogenetic utility of different regions (Li et al., 2013; Evans, Joshi & Wang, 2019). It is clear that the incongruent relationships resulted from different partition data sets. In the present study, three partition data revealed different topologies compared to 75 protein-coding genes (Fig. 3; Figs. S2–S4). However, these data support the monophyly of *Cenchrus*, suggesting the sufficiency of the phylogenetic utility of different chloroplast genome regions at genus level in Poaceae.

### The efficiency of SNP-based molecular markers for *Cenchrus longispinus*

A molecular marker is a useful tool for exploring genetic variations, identifying traits, and distinguishing species (Hayward et al., 2015; Garrido-Cardenas, Mesa-Valle & Manzano-Agugliaro, 2018). In Poaceae, various molecular markers have been developed (Liu et al., 2012; Hammami et al., 2014; Gao, Jia & Kong, 2016). For example, traits in important species of Poaceae (i.e., bamboo, and wheat) can be traced using the SNP markers (Jiang, Zhang & Ding, 2013; Hu et al., 2015; Przewieslik-Allen et al., 2019). Besides that, plastid data were used for species discrimination of *Stipa* (Krawczyk et al., 2018). Previously, although there are reports on the complete cpDNA sequences of *Cenchrus*, there have no studies on developing markers for *Cenchrus* (Bhatt & Thaker, 2018; Wu & Zhou, 2018). Being classified as an invasive alien plant, more data of *C. longispinus* should be collected. In this study, we employ the SNP sites in *C. longispinus* to develop molecular markers which can be used under the same PCR protocol (Table S3). These newly developed markers will be useful for identifying *C. longispinus* during its invasion in Korea. The information on invasive patterns of *C. longispinus* will be useful to make profound strategies for control its invasion in Korea. Previously, the hybrids between *Cenchrus* and related species were identified and characterized (Read & Bashaw, 1974; Marchais & Tostain, 1997; Quiroga et al., 2013). Therefore, the present genomic data will be essential for further identifying the hybrid between *C. longispinus* and related species. Addition to the SNP sites, the SSR data were used to develop molecular markers for different species in Poaceae (Hodkinson, De Cesare & Barth, 2013; Jiang, Zhang & Ding, 2013; Hammami et al., 2014). In this study, although the SSR markers were not developed, the availability of SSR in the cpDNA of *C. longispinus* will provide fundamental information for further studies on both population genetics and SSR-based molecular markers in Poaceae.

## Conclusions

This study provides the first complete chloroplast genome sequences of *C. longispinus* and *C. echinatus* which can be used for further studies on population genetics, phylogeny, and comparative genomics of *Cenchrus* in particular and the family Poaceae in general. Also, the newly developed molecular markers based on SNP sites and simple PCR protocol can be useful for the identification and management of the invasion of *C. longispinus* in Korea.

## Supplemental Information

10.7717/peerj.7965/supp-1 Supplemental Information 1 The PCR results of specific primer pairs for *Cenchrus longispinus*. (A) The specific primer pairs for *atpB* gene; (B) The specific primer pairs for *matK* gene; (C) The specific primer pairs for *ndhD* gene. The number from one to 10: *Cenchrus longispinus*; from 11 to 20: *Cenchrus echinatus*; from 21 to 30: *Pennisetum alopecuroides*.

10.7717/peerj.7965/supp-2 Supplemental Information 2 The Maximum likelihood tree of *Cenchrus* and related taxa inferred from LSC region. The numbers mean supporting values. LEC, Lecomtelleae; PAN, Paniceae; PAS, Paspaleae; Pasp, Paspalinae; Anth, Anthephorinae; Boiv, Boivinellinae; Neur, Neurachninae; Dich, Dichantheliinae; Pani, Panicinae; Meli, Melinidinae; Cenc, Cenchrinae.

10.7717/peerj.7965/supp-3 Supplemental Information 3 The Maximum likelihood tree of *Cenchrus* and related taxa inferred from SSC region. The numbers mean supporting values. LEC, Lecomtelleae; PAN, Paniceae; PAS, Paspaleae; Pasp, Paspalinae; Anth, Anthephorinae; Boiv, Boivinellinae; Neur, Neurachninae; Dich, Dichantheliinae; Pani, Panicinae; Meli, Melinidinae; Cenc, Cenchrinae.

10.7717/peerj.7965/supp-4 Supplemental Information 4 The Maximum likelihood tree of *Cenchrus* and related taxa inferred from IR region. The numbers mean supporting values. LEC, Lecomtelleae; PAN, Paniceae; PAS, Paspaleae; Pasp, Paspalinae; Anth, Anthephorinae; Boiv, Boivinellinae; Neur, Neurachninae; Dich, Dichantheliinae; Pani, Panicinae; Meli, Melinidinae; Cenc, Cenchrinae.

10.7717/peerj.7965/supp-5 Supplemental Information 5 Supplemental Tables.

10.7717/peerj.7965/supp-6 Supplemental Information 6 The chloroplast sequence of *Cenchrus longispinus*.

10.7717/peerj.7965/supp-7 Supplemental Information 7 The chloroplast genome sequence of *Cenchrus echinatus*.

## Additional Information and Declarations

### Field Study Permissions

The following information was supplied relating to field study approvals (i.e., approving body and any reference numbers): The destruction of samples from specimens was approved by Korea National Arboretum (KNA), Gachon University Herbarium (GCU), The University of Texas at Austin Herbarium (TEX), and The Queensland Herbarium (BRI).

### Data Availability

The following information was supplied regarding data availability: *Cenchrus longispinus* and Cenchrus echinarus data are available at GenBank: MN078361 and MN078360.
