# Correction: Development of molecular markers for invasive alien plants in Korea: a case study of a toxic weed, *Cenchrus longispinus* L., based on next generation sequencing data

**DOI:** 10.7717/peerj.7965/correction-1

**Published:** 2019-12-16

**Authors:** JongYoung Hyun, Hoang Dang Khoa Do, Joonhyung Jung, Joo-Hwan Kim

**Affiliations:** Department of Life Science, Gachon University, Seongnam, Gyeonggi, Korea

Corrigendum: 

After publication, the authors noticed an error in the authorship of the scientific name of *Cenchrus longispinus* and incorrect usage of the word “toxic” for *C. longispinus* in the title. The title is now written as: “Development of molecular markers for invasive alien plants in Korea: a case study of a noxious weed, *Cenchrus longispinus* (Hack.) Fernald, based on next generation sequencing data”.

Additionally, the species name *Cenchrus echinatus* is now italicized in the Data Availability statement.

Original PDF

