# Supplementary figures and images for: Development of molecular markers for invasive alien plants in Korea: a case study of a toxic weed, Cenchrus longispinus L., based on next generation sequencing data
Source: PeerJ. 2019 Nov 11;7:e7965. doi: 10.7717/peerj.7965 (PMC6855208; doi:10.7717/peerj.7965)

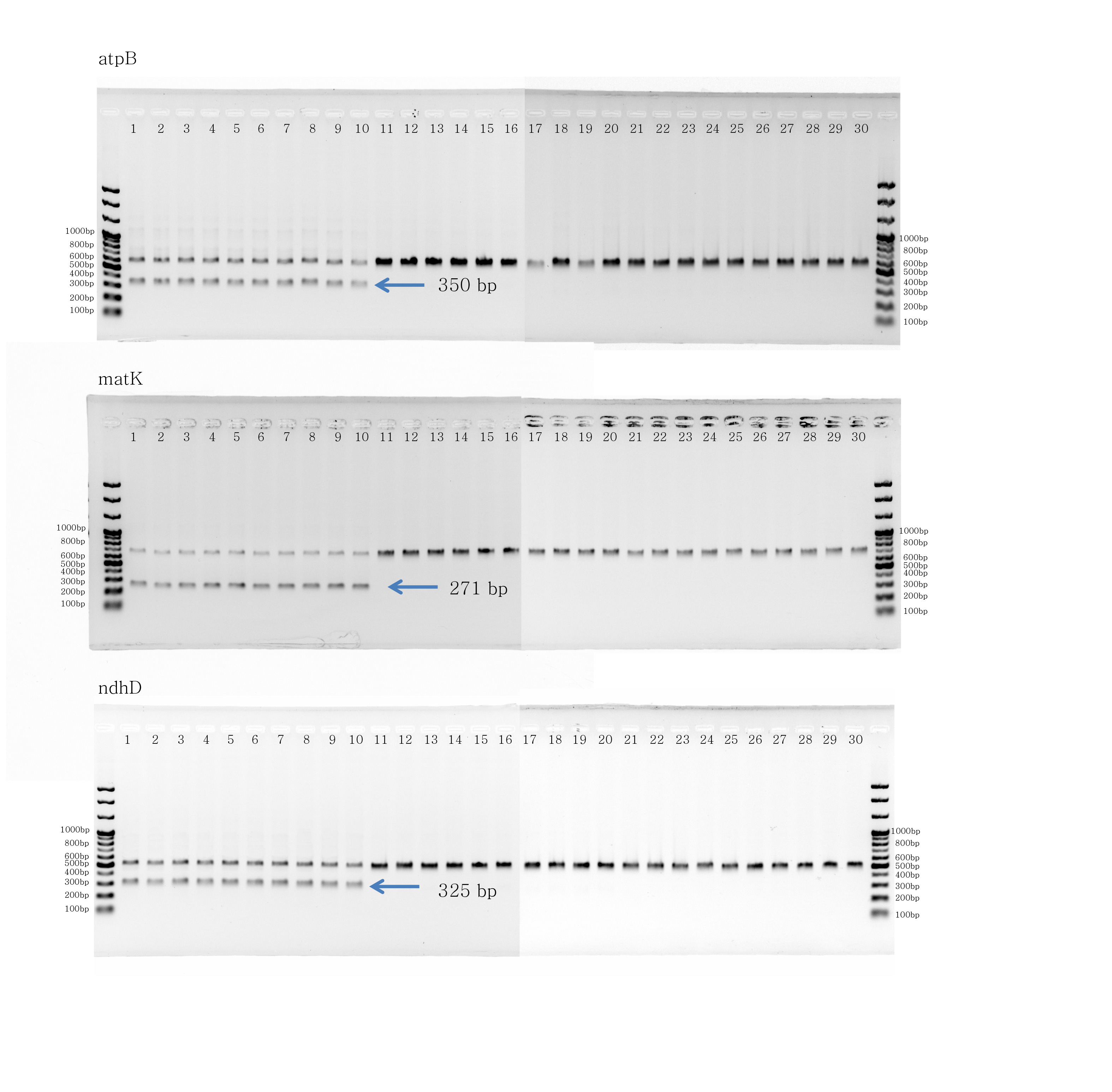

Supplement: Supplemental Information 1 — (A) The specific primer pairs for atpB gene; (B) The specific primer pairs for matK gene; (C) The specific primer pairs for ndhD gene. The number from one to 10: Cenchrus longispinus; from 11 to 20: Cenchrus echinatus; from 21 to 30: Pennisetum alopecuroides. [file peerj-07-7965-s001.jpg]

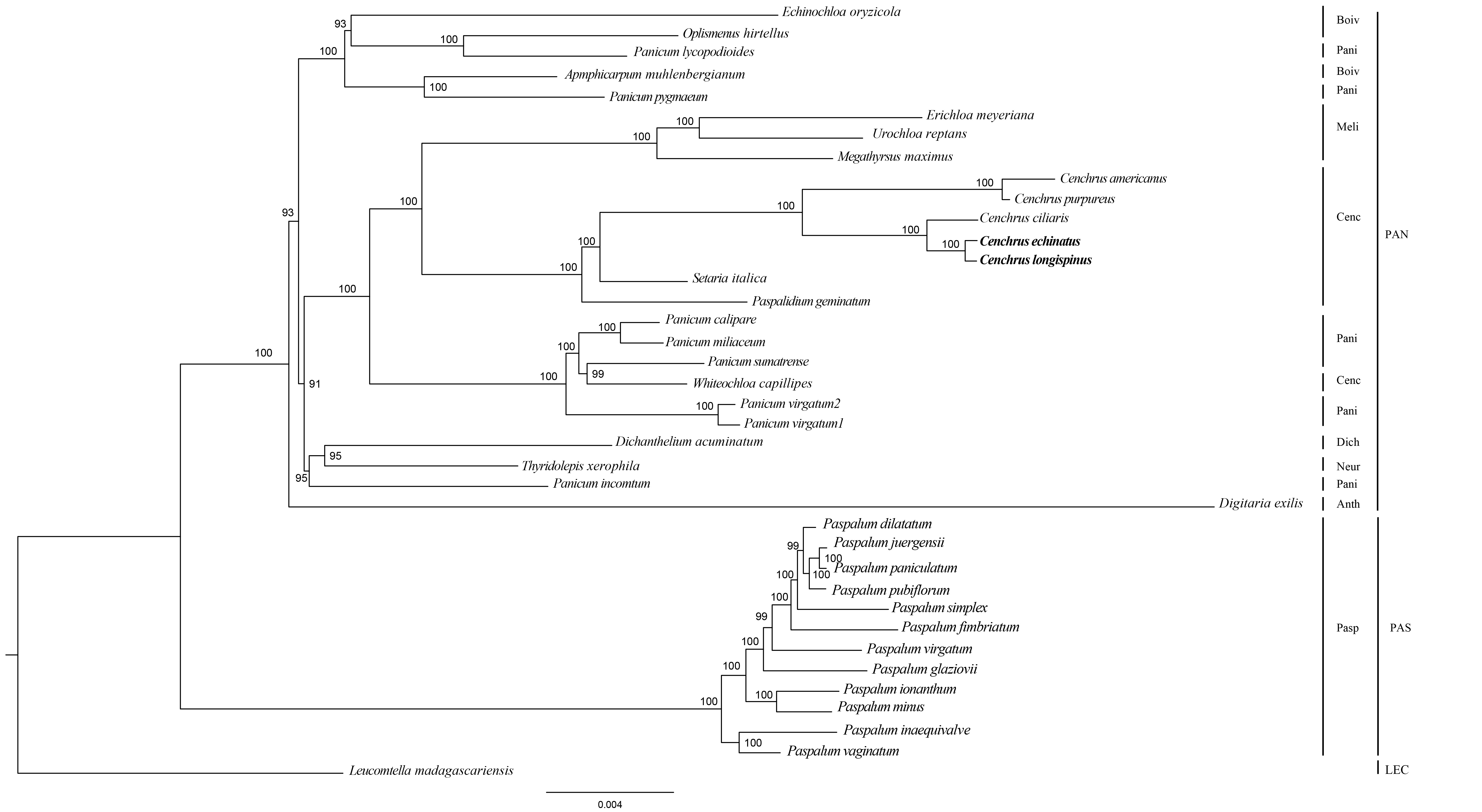

Supplement: Supplemental Information 2 — The numbers mean supporting values. LEC, Lecomtelleae; PAN, Paniceae; PAS, Paspaleae; Pasp, Paspalinae; Anth, Anthephorinae; Boiv, Boivinellinae; Neur, Neurachninae; Dich, Dichantheliinae; Pani, Panicinae; Meli, Melinidinae; Cenc, Cenchrinae. [file peerj-07-7965-s002.jpg]

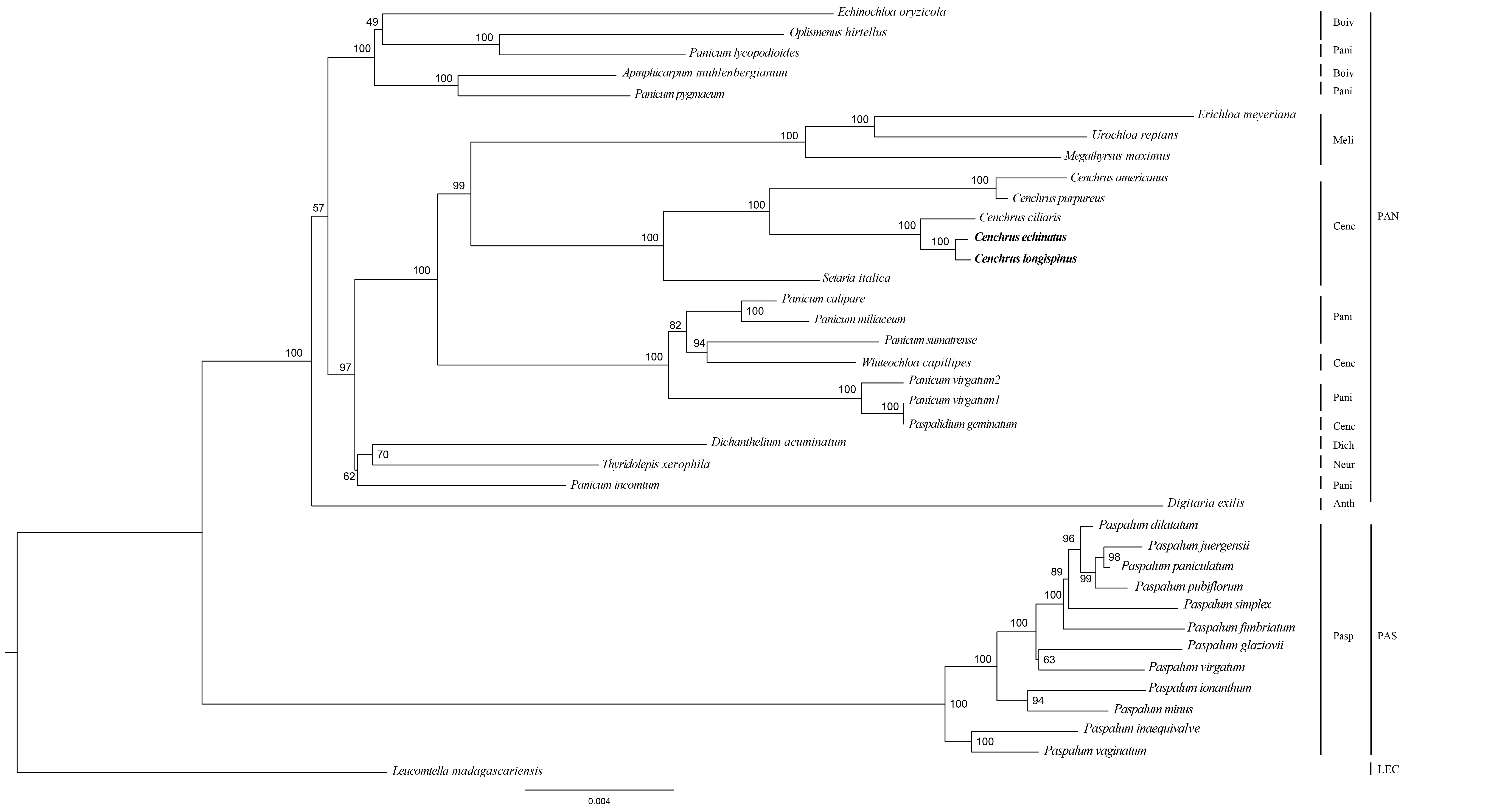

Supplement: Supplemental Information 3 — The numbers mean supporting values. LEC, Lecomtelleae; PAN, Paniceae; PAS, Paspaleae; Pasp, Paspalinae; Anth, Anthephorinae; Boiv, Boivinellinae; Neur, Neurachninae; Dich, Dichantheliinae; Pani, Panicinae; Meli, Melinidinae; Cenc, Cenchrinae. [file peerj-07-7965-s003.jpg]

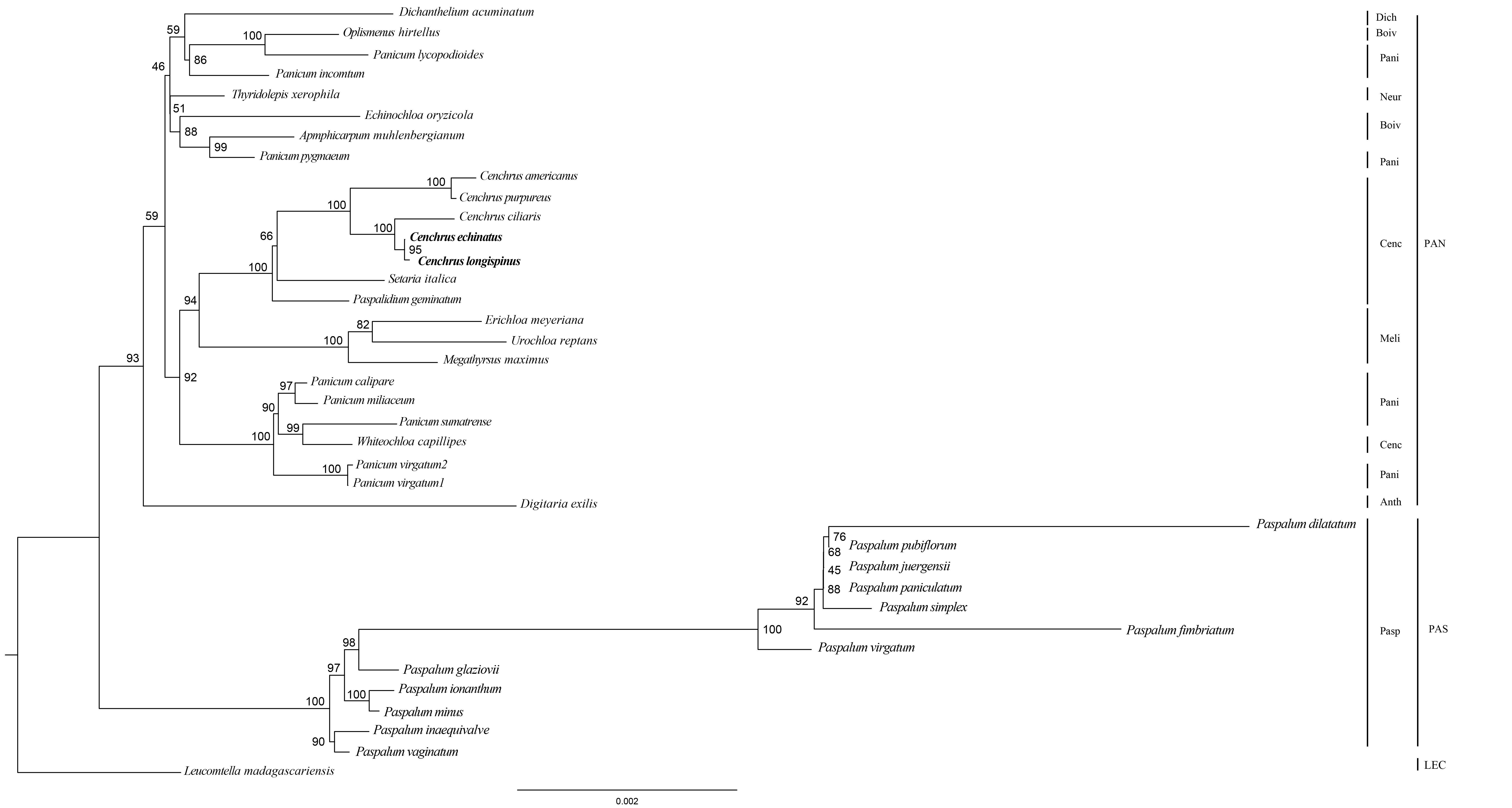

Supplement: Supplemental Information 4 — The numbers mean supporting values. LEC, Lecomtelleae; PAN, Paniceae; PAS, Paspaleae; Pasp, Paspalinae; Anth, Anthephorinae; Boiv, Boivinellinae; Neur, Neurachninae; Dich, Dichantheliinae; Pani, Panicinae; Meli, Melinidinae; Cenc, Cenchrinae. [file peerj-07-7965-s004.jpg]
